# An Account of a New Mode of Preserving an Equable and Salutary Temperature of the Air of Rooms for Consumptive Patients

**Published:** 1821-07

**Authors:** Thomas Andrew Knight

**Affiliations:** President of the Horticultural Society, &c. &c.


### *An Account of a new Mode of preserving an equable and salutary Temperature of the Air of Rooms for Consumptive Patients.* By Thomas Andrew Knight, Esq. F.R.S. President of the Horticultural Society, &c. &c.

I TRANSMIT the following communication to the London Medical and Physical Journal, under an impression that it points out a better method of creating an artificial climate for patients with tender lungs, than any generally known; and I write with the hope of averting from other parents, and other families, a calamity which I believe it has been the means of averting from me and my family.

The patient, who was the subject of the mode of treatment which I proceed to describe, had been perfectly healthy, though her appearance was delicate, from her birth, till she married, and had one child. Her appearance then, in her nineteenth year, became consumptive; and, within a few weeks afterwards, a sudden and very considerable discharge of blood, apparently from her lungs, but too plainly pointed out the nature of her complaint. It was proposed immediately to convey her to a southern climate; but, owing to events which I need not detail, this was not done. She passed the first winter in rooms which were kept warm and of as equal temperature as circumstances would permit; but there was a necessity for several blisters and the application of leeches. In the succeeding summer, her health and strength appeared somewhat to improve; but at the end of the following winter she was so much emaciated and reduced by a long-continued discharge of blood from her lungs, and the effects of her feverish state, that I thought all hope of her recovery past; and I should have been much less unhappy respecting her if she had been at peace in her grave. I had, however, the consolation to find that her physician, Dr. Wilson Philip, (whose important and singular physiological discoveries have, of course, made his name familiar to every medical man,) did not despond. He pronounced decisively that, if she remained another winter in the climate of England, her death was inevitable; but he expressed his hope and belief that, if she could be placed in a situation where she could have the benefit, during eighteen successive months, of a climate as favourable as the best part of an English summer, she would recover her health and strength, and be subsequently able to bear an English winter, though her lungs could never wholly regain their former power. Measures were consequently taken to convey her abroad; but I found in her a settled conviction that she should go never to return, and that her remaining strength was wholly unequal to the exertion necessary.

Under these circumstances, I thought only of the best means of giving her, as far as practicable, the advantages of a warm and temperate climate at home; and, having been much in the habit of making such climates for plants, I looked forward with some hope, though with trembling anxiety, to the result. I stated to Dr. Philip my confidence that I could give to the air of the rooms in which I proposed that she should pass the winter, such a degree of humidity (she breathed most freely in somewhat damp weather,) as should be found best to agree with her, and any temperature, with little variation, which he should think likely to be most beneficial; and the following plan was adopted, with (under existing circumstances,) his most unqualified approbation. A flue of sufficient power, and surrounded by an air-chamber of just sufficient dimensions to permit a person to go round the flue to ascertain that no smoke escaped into the air-chamber, was constructed wholly of brick-work; space being prepared to receive garden-pots, to be filled with wetted sand, to give to the heated air the requisite degree of humidity; and, from the top of this air-chamber, pipes of tin, cased with wood, were made to convey a warm current of air, to rise through different parts of the floor of every room into which she should have occasion to enter. She was thus given the benefit of a warm temperature,—that of sixty degrees, with little variation,—as proposed by Dr. Philip, with a constant and rapid change of air. A summer temperature of eighteen months was thus given, and the result has succeeded my most sanguine hopes. The patient has perfectly recovered her health and strength, and she suffers no further inconvenience from her past illness than inability to walk briskly up hill, or to sustain any continued bodily exertion, without being soon out of breath.

Particular attention was paid to prevent the temperature of the air of the bed-room of the patient from becoming too low at an early hour of the morning in winter; that being the time at which, I have had reason to believe, patients with tender lungs usually suffer most in cold weather. It is probable that, in the case above stated, the patient was, to a great extent, benefitted by judicious medical treatment in every other respect: but upon this subject I do not feel qualified to speak.

*London*; *June* 13, 1821.

